# Sharing or Not: Psychological Motivations of Brand Rumors Spread and the Stop Solutions

**DOI:** 10.3389/fpsyg.2022.830002

**Published:** 2022-04-04

**Authors:** Xu Zhang, Hong Zhu, Yu Huang, Chunqu Xiao

**Affiliations:** ^1^Business School, Nanjing University, Nanjing, China; ^2^Business Administration School, Shanxi University of Finance and Economics, Taiyuan, China

**Keywords:** brand rumors, consumer cognition, sharing rumors motivation, social contagion theory, fsQCA

## Abstract

Brand rumors can harm brands’ image and bring significant impacts on customers’ decision-making and sharing behavior. Finding practical strategies for preventing the spread of brand rumors continues to be a challenge. Building on the social contagion theory, the current research enriches the discussion on understanding why people spread rumors and how to deal with the spreading of rumors. Sharing brand rumors is motivated by a variety of complex psychological reasons, but prior research didn’t adequately analyze the problem from a complexity perspective. Therefore, using a sample of 416 interviewers within eight types of brand rumors, this study employs fuzzy-set qualitative comparative analysis (fsQCA) to investigate the combination of rumor psychological communication motivations in brand activities and solutions to prevent the spread of brand rumors. The current study discoveries three and two first-level configurational solutions, respectively, that can promote positive and negative rumor spreading. To summarize, emotional stimulation is a key component in the spread of rumors; altruism and relationship management motivation can coexist at times; and untrusted rumors are disseminated through other motivation factors. Solutions to prevent rumors from spreading are also provided. Furthermore, the findings help to understand the psychology of configurational motivation and how it can help brands reduce the spread of brand rumors. Finally, these discoveries’ theoretical contributions and practical implications are presented.

## Introduction

Although no one trusts rumors, people believe in “facts” (Fine, 2007). Rumors significantly impact customers and brands because of their extensive spread and numerous influences. First, rumors that surface under the pretext of the “truth” mask surround customers and become consumers’ shopping basis; this influences their purchasing decisions, causing economic losses. Evidence shows that when consumers lack theoretical knowledge and critical thinking ability, rumors and options provided by those around them influence their decision-making, and this causes a herd mentality. Rumors can provide reasonable explanations for uncertain or ambiguous situations (DiFonzo et al., 1994; Bordia and DiFonzo, 2002). Therefore, due to anxiety, consumers are more likely to follow the choices made by crowds to gain a sense of security and avoid the risk of making the wrong decision; as a result, they are more likely to make blind purchasing decisions (Loxton et al., 2020).

Second, brands compelled to be associated with rumors frequently appear passive and overwhelmed. *This is because the consumer loyalty and brand image have contributed to high product sales and market share (Kovacova and Lewis, 2021), but some research indicates that brand rumors impact brand image, sales share, and brand satisfaction and loyalty (Kimmel, 2004; Kimmel and Audrain-Pontevia, 2010).* Negative rumors negatively impact the brand (Kimmel and Audrain-Pontevia, 2010). For example, rumors about KFC’s mutant chickens with 8 wings had a major blow to the market share and brand image of the company. Although the final rumormongers were ordered to pay KFC $91191, this could not offset the economic losses caused by the rumor. Scholars ignore the positive brand rumors, which have a complex influence but undoubtedly harm consumer interest. While positive rumors boost the market share of a brand, customers who buy the goods lose interest once the rumors are proven to be false, thereby damaging the brand value (Aditya, 2014). Whilst positive rumors may promote a brand to gain long-term market share, the market share of some brands quickly fall into a trough after a short-term surge, particularly when rumors are exposed as false. For instance, although studies have demonstrated that ginger shampoo is ineffective (Miao et al., 2013), this well-known care brand has continued to sell well because the idea that ginger is good for hair growth is deeply ingrained in consumers’ minds. In early 2020, Shuang-Huang-Lian (a type of Chinese herb) was rumored to prevent COVID-19, prompting consumers to rush to buy it; however, 3 days later, the news was pronounced as fake. Although the Shuang-Huang-Lian company did not create this rumor, the turmoil caused a rapid fall in the company’s stock price to the bottom after three consecutive days of rising. Current research on the strategies’ companies adopt to prevent the spread of brand rumors is scarce, causing loss of brands due to rumor infringement. Therefore, this study looks at the psychological path of consumers who spread rumors and investigates the psychological path analysis to prevent the spread of rumors. The findings will provide more approaches for companies to maintain their brands when major rumors arise, and offer specific guidance and reference for healthy brand development.

Individual participation is inextricably linked to the spread of rumors. Individuals serve as the information transmission box, spreading rumors in the dissemination process. Rumors are defined as using simple associations or assumptions to create and disseminate different or contradictory statements to facts (DiFonzo and Bordia, 2007). Individuals sharing rumors essence is a group behavior characterized by the dissemination of information. Contrary to the idea that original rumors spread face-to-face, the number, spread speed, and scope of influence of online rumors are constantly expanding in the internet age due to the rapid advancement in internet technology and the continuous growth of social media users (Bloch et al., 2017). However, the ease with which information may be accessed and transmitted through social media has resulted in ambiguity, misinformation, and doubt (Sharma and Kapoor, 2022). Moreover, internet rumors are anonymous, interactive, and free of charge; this reduces the spread of psychological and material costs and promotes the spread of rumors (Difonzo, 2013). In this view, the present study primarily focuses on internet rumors with a wide range of influences on consumers.

While rumors are certainly a societal phenomenon, psychological reasons at an individual level are a critical component of the rumor puzzle (Bordia and Di Fonzo, 2017). Previous studies lacked a comprehensive examination of the psychological motivations of individuals to spread rumors from a holistic standpoint. Current exploration on the primary psychological motivations behind rumor spreading focuses on the net effect of passive psychological factors, including information uncertainty, anxiety, conformity, and gullibility (Donovan, 2003; Difonzo, 2013; Duffy et al., 2020). These investigations are geared toward the net effect of psychological motivation elements. The scholars ignore that people’s psychological motivation to spread rumors is a multifaceted effect associated with the internal and external environment, and personal characteristics. Furthermore, few scholars recognize the contribution of rumor psychology spreading in marketing strategy (Kimmel and Audrain-Pontevia, 2010). Therefore, to protect the interests of consumers and brands, the present work investigates the combined psychological path of rumors spreading and unravel approaches to prevent the spread of brand rumors under various circumstances. The analysis is based on previous research on stimulating the psychological communication factors of rumors.

The theoretical significance of the present study is reflected in four aspects. First, this research significantly theoretically contributes to the sharing and dissemination of rumors. It thoroughly investigates and compares the psychological communication motivations for positive and negative brand rumors, thereby filling the gap in previous research that focuses primarily on negative brand rumors but ignores the psychological path taken by positive brand rumors. Second, considering the cognitive evaluation theory, this study investigates the rumor spreading and prevention psychology pathways, exploring the overall effect of multiple psychological stimuli. Third, the present investigation has some practical implications; for instance, when the brand encounters major rumors, this work explores strategies to maintain brands, providing guidance and reference for healthy brand development and reducing the economic loss and reputation loss of brands. Fourth, unlike traditional statistical methods for investigating the net effect, the present study employs fuzzy-set qualitative comparative analysis to evaluate the combined impact of psychological motivation to stimulate rumor spreading, opening up avenues for brands to assist in rumor prevention.

## Literature Review

### Social Contagion Theory

The term’ social contagion’ was coined by Redl (1949). Scholars have the view that individual cognition and behavior are contagious within a group or society, and particular behavior and cognitions of members of a group continuously change as a result of their environment and interactions with others (Bandura, 1986); this results in contagious social behavior (Redl, 1949). According to social contagion theory, individual and group factors and their interactions must be accounted for when evaluating contagious social behaviors (Redl, 1949; Turner and Killian, 1957). Social contagion behavior refers to the spread of emotions, attitudes, cognition, and behavior from “initiator” to “recipient” (Wheeler, 1966). Social contagion behavior facilitates the transmission of information, emotion, and behavior from the initiator to the receiver during the contagion process (Burt, 1987). Invariably, some recipients in social networks nearly accept information and behavior diffused by initiators who are motivated either by active or passive psychological motives during dissemination (Redl, 1949).

Social contagion theory examines the impact of the social environment on the attitudes and behaviors of an individual and the internal mechanism of social contagion between individuals and groups (Burt, 1987). Numerous studies have demonstrated that individual and group factors are the primary factors of social contagion behavior; however, their interaction triggers social contagion events (Barsade, 2002). Also, studies on the internal logic of the two interactions are rare (Redl, 1949). Therefore, the present research addresses this research gap in conjunction with the research questions about rumor spreading.

### Brand Rumor Spreading and Social Contagion Theory

*A brand is no longer defined by what we tell consumers; rather, it is defined by what customers tell one another (Gavurova et al., 2018)*. Thus, as socially contagious events, group interaction with individual factors should be considered when evaluating rumors spread within consumer groups. Depending on various situations and individual differences, rumor spreading has varying effects on infectious social events (Levy and Nail, 1993). *While technologies such as data-driven predictive algorithms are helping to manage online rumors, the psychological motivations of those spreading rumors should be addressed (Kovacova and Lewis, 2021).* As a form of contagious event behavior, rumor spreading mainly necessitates the establishment of distinct channels of communication between individuals and groups (Dubois et al., 2011).

The initiator of the behavior has distinct transmission paths based on their cognitions and motivations (Mitchell, 1982). Psychological motivations for rumor spreaders are primarily active and passive. First, considering passive psychology, individuals spread rumors from three distinct emotional, cognitive pathways: positive, negative, or herd mentality. Evidence shows that different psychological motivation models have varying effects on the manner and content of group rumors (Reeve, 2005). Spreading rumors based on passive psychological stimulation is primarily motivated by the emotional cognition of an individual (Lemerise and Arsenio, 2000; Waddington and Fletcher, 2005). Some studies have revealed that emotional contagion is the process whereby an individual or group influences another by inducing emotional states, attitudes, and behaviors (Schoenewolf, 1990). Emotional cognition transmits emotional information to the cognition of others based on their circumstances, consequently influencing their cognition and behavior (Van Kleef, 2009, 2016; Van Kleef et al., 2015). Additionally, rumor dissemination motives based on active psychology influence how rumors are transmitted due to their internal needs, including altruistic behavior, self-enhancement, and relationship management motivation.

In this view, according to social contagion theory, this study, by combining consumer motivational psychology with the transmission and reception pathway of rumors, deeply analyses the relationship between the configuration path of the interaction mode between consumers and groups and the relationship between positive and negative rumors. The research findings suggest solutions for businesses to prevent the spread of brand rumors.

### Motivation for Passively Sharing Rumors

Rumors may be created intentionally or inadvertently and spread by individuals. According to the findings of Allport and Postman (1947), the primary motivation for the spread of rumors stems from the ambiguity and importance of the rumor to people. The ambiguity of rumors deepens as they spread. On the other hand, activating certain psychological factors, including anxiety (Rosnow, 2004) or hope (Esposito, 1987), promote people’s perception of the importance of rumors. Moreover, studies have revealed other variables associated with rumors spread research, including the herd mentality caused by the spread of large-scale rumors (Bordia and DiFonzo, 2004; Fine, 2007), involvement (DiFonzo and Bordia, 2007), and psychological control (DiFonzo and Bordia, 2007). The following sections describe the contributions of people’s passive psychological motivations, such as anxiety, hope, and herd mentality, to the spread rumors.

#### Anxiety Management Motivation With Sharing Rumors

Anxiety is one of the significant factors for rumor dissemination at the psychological stage of the distribution of rumors (DiFonzo and Bordia, 2007). Uncertainty impairs individuals’ ability to cope with their environment effectively, eliciting feelings of helplessness and anxiety. DiFonzo and Bordia (2002) found that highly anxious subjects can spread rumors with less stimulus than non-anxious individuals. Sharing rumors is a successful strategy to alleviate anxiety for individuals who cannot clarify or lack accurate facts (DiFonzo and Bordia, 2007). Anxiety is a kind of negative emotional state developed when one worries about potential outcomes. A previous meta-analysis by Rosnow (1991) of seven rumors revealed a strong average linear impact (*r* = 0.48) on the relationship between fear and spreading rumors. In addition, for individuals with little information or messages about the truth, rumor-spread disinformation can motivate incorrect decision making or cause economic losses.

#### Hope Management Motivation With Sharing Rumors

Hope is a type of positive emotional state based on the pathway of successful planning (Snyder et al., 1991). Emerging evidence indicates that hope is relevant to people’s actions (Kemp et al., 2017). Brand rumors that provide consumers with hope are accompanied by optimism and possibly increase consumer purchase intent (Miceli and Castelfranchi, 2010). Especially, positive messages (maybe rumors) in marketing and advertising frequently imply that consumers will become more beautiful, healthy, younger, or stronger as a result of using the product, regardless of whether this is true (Kemp et al., 2017). Rumors of hope invoke an anticipated repercussion, prompting customers to believe and behave according to the content of the rumors. Hope is linked to the spread of medical, beauty, and wellness rumors (Chua, 2015). People believe and consider the realness of hope rumors because hope can alleviate tension and negative feelings (Miceli and Castelfranchi, 2010).

#### Herd Mentality With Sharing Rumors

People develop herd mentality, a naturally formed unified action or thinking, to escape social strain (Loxton et al., 2020). As social beings, people typically have “social” behavioral characteristics. Herd mentality demonstrates succumbing to the strain of communities (Crutchfield, 1955). The wishes of individuals have to obey common choices because residents recognize that their deviation from social norms may significantly harm their social standing. Therefore, retaining a group attitude and behavior (like a herd) is a strategy to decrease personal risks in the face of group pressure. The theory put forward by Fine (2007) indicates that people tend to follow the crowd to escape failure or punishment, especially when they lack critical thinking abilities or appropriate information. The herd mentality effect is associated with the widespread of rumors, prompting consumers to make stupid buying decisions.

### Motivation for Actively Sharing Rumors

Apart from passively spreading rumors, research has revealed that people are motivated to actively spread rumors (DiFonzo and Bordia, 2007; Bordia and Di Fonzo, 2017; Sudhir and Unnithan, 2019). Aside from motivation to find the truth because of uncertainty, Bordia and Di Fonzo (2017) emphasized the motivation to actively spread rumors from the social interchange service. The main components essential for good social interchange are as follows: acting effectively, building relationships and self-improvement, and the ability to predict each other’s intentions or behaviors (Haroush and Williams, 2015; Bordia and Di Fonzo, 2017). Acting effectively is related to trust in a manner that people collect information to make decisions. It is also imperative to establish and maintain interpersonal relationships in society. Regarding self-improvement, people tend to validate their self-awareness and boost their self-esteem in various ways using the social system. Moreover, altruism contributes significantly to the spread of rumors when perceived as helpful information (Apuke and Omar, 2021).

#### Trust and Sharing Rumors

Trust is a critical mechanism of social operation and a vital component of the synthetic power of a society. Trust influences consumer behavior. Rumors and trust are inseparable in the study of brand rumors. Studies show that rumors influence individual beliefs, attitudes, and behaviors (Bordia and Di Fonzo, 2017) because rumors spread like “true information” before they are verified as rumors. Research evidence indicates that people who trust rumors are more likely, intentionally or accidentally, to spread rumors (Fine, 2007; Chua and Banerjee, 2018). Additionally, people share rumors to discover the truth and reduce feelings of lack of control and anxiety when they are unsure whether rumors are true or false (Bordia and Di Fonzo, 2017).

Additionally, various factors in social media influence the establishment of user trust. First, consumer trust in brands impacts the spread of brand rumors. A high level of brand trust among consumers reduces doubts and inferences about positive rumors regarding a brand; instead, consumers regard this as positive brand information to promote spread. Negative rumors about a brand can be supplemented with counter-arguments and online debates (Amine, 1998). Second, users can doubt the credibility of brand rumors due to the anonymity of online rumors. However, the likelihood of consumers believing them and joining the propagation team increases when social, interpersonal relationships surrounding consumers spread rumors (Wang et al., 2021).

#### Relationship Management Motivation and Sharing Rumors

Relationship management motivation refers to customers sharing rumors when they find it beneficial to improve their relationship with other consumers (DiFonzo and Bordia, 2007). Generally, the reason for spreading rumors is to reap their benefits (DiFonzo and Bordia, 2007). Consumers share “information” (rumors) to other consumers for closer connections. If consumers think that the “information” can bring losses to other consumers, they distribute it broadly to prevent losses. In contrast, if consumers feel that the “information” can profit others, they share it with other consumers, helping them achieve benefits and strengthen relationships. The sharers and recipients of rumors converse and communicate during the “information” distribution process in order to increase the likelihood of developing long-term relationships (DiFonzo and Bordia, 2007).

#### Self-Improvement Motivation and Sharing Rumors

Self-enhancement motivation shared by rumors refers to the motive by consumers to relay rumors to another customer to increase self-confidence and self-esteem (DiFonzo and Bordia, 2007). Self-enhancement motivation emphasizes the need to feel good in sharing rumors, that is, the cognitive processing of rumors. People may post and condemn the rumors if the substance of the rumors contradicts their beliefs to boost their confidence. When people think that the rumor concurs with their values, they strengthen their ideas and become willing to believe that the rumor is true. This boosts their self-image and self-esteem when customers use “information” (rumors) to promote their favorite brands. Word-of-mouth contact refers to non-commercial communicators who disseminate knowledge or evaluations to other consumers or potential consumers regarding goods, brands, organizations, and services (Sudhir and Unnithan, 2014). Businesses hope that customers provide their brand with positive word-of-mouth communication as one strategy to quickly increase market share. Therefore, some rumors are circulated by the merchants themselves to advertise the product. However, this motivation is rare in the research on the spread of brand rumors (Sudhir and Unnithan, 2014).

#### Altruism and Sharing Rumors

Altruism is a type of motivation that seeks to benefit others (Batson, 2010). In the context of information transmission, altruism refers to sharing information with others, expecting nothing in return (Ma and Chan, 2014). The notion of altruistic individuals serving others implies that they have an incentive to spread rumors whenever they are deemed valuable information (Ma and Chan, 2014; Plume and Slade, 2018). Altruism rumor sharing motivation refers to individual motivation to trade rumors to assist others (Apuke and Omar, 2021). Recent research shows that people are willing to share information to assist others, regardless of whether the information is true or false; individuals can take precautions to avoid losses based on this information (Destiny Apuke and Omar, 2020). Apuke and Omar (2021) revealed that individuals with a higher level of altruism are more likely to spread misinformation about COVID-19 and attempt to share helpful information with others. As such, one might anticipate a connection between altruism and rumor-sharing behavior.

**FIGURE 1:**
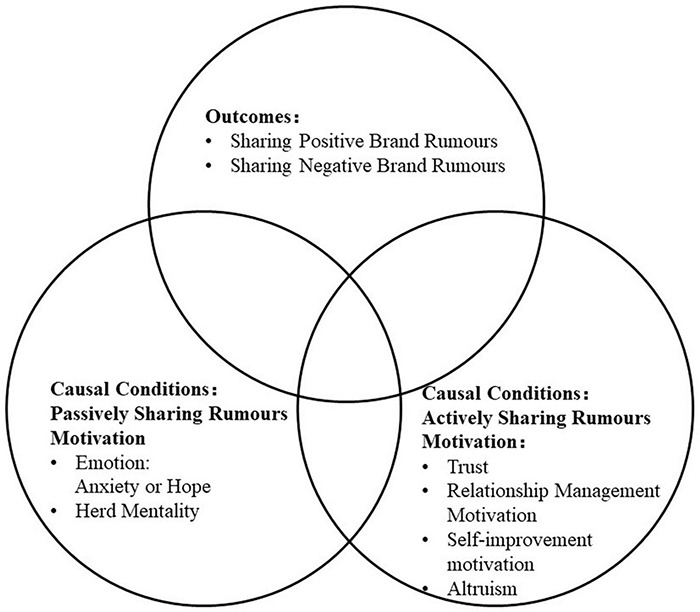
The conceptual model of sharing positive or negative brand rumors.

In conclusion, the present research on the psychological motivation of rumors focuses on the passive psychological motivation influenced by external factors to share the rumors impulse (Difonzo, 2013; Wang et al., 2019; Duffy et al., 2020), the active sharing motivation based on their requirements (DiFonzo and Bordia, 2007), and personal cognitive level of trust in rumors (Ma, 2008; Difonzo, 2013; Duffy et al., 2020). However, the sole focus on the net effect of one or more psychological factors on individuals sharing rumors ignores the fact that various factors influence the spread of individual rumors; this cannot fully explain the complex psychological motivations of individuals sharing rumors. Furthermore, only a few studies have explored the psychological communication motivation of positive or negative brand rumors. To fill the gap, the present article examines psychological motivations for rumor spread configurations from the perspectives of passive psychological motivation, active psychological motivation, and personal cognitive level of trust in rumors, and the approaches to stop brand rumors from spreading in different situations. The conceptual model of this research, which is based on the literature review, is outlined in Figure 1.

## Methodology

### Overview of Fuzzy-Set Qualitative Comparative Analysis

Fuzzy-set qualitative comparative analysis (fsQCA) is a qualitative and quantitative research method different from traditional linear correlation quantitative analysis. This approach supports method mining for non-linear and asymmetric relationships (Rihoux and Ragin, 2012). The fsQCA studies address the complex causal hypotheses based on essential and sufficient conditions (Schneider and Wagemann, 2006). By comparing business research methods from Woodside (2013), fsQCA is an alternative data analysis and theory creation method. fsQCA is founded on configuration theory (Greckhamer, 2011) which employs the Boolean algebra rule for operations and focuses on element interaction, joint causes, and configuration effects (Ordanini and Maglio, 2009; Morgan, 2010). Furthermore, fsQCA is appropriate for measuring the essential and sufficient conditions for a specific result, particularly when multiple factors contribute to the outcome (Rihoux and Ragin, 2012). Also, it provides high-performance and non-high-performance detection for causal asymmetry (Fiss, 2011). According to the set theory and complexity theory, fsQCA technology focuses on “multiple concurrent causalities” across cases; in this view, the same result can be obtained by multiple combinations of elements (Ragin, 2013). Thus, fsQCA is receiving extensive use in management and consumer behavior research, which has to contend with a complex environment (Mikalef, 2017; Cruz-Ros et al., 2018; Kraus et al., 2018; Kaya et al., 2020; Phung et al., 2020). The present work explored the psychological factors and combinations of psychological factors encouraging individuals to spread brand rumors in the complex social environment. Therefore, fsQCA 3.0 was employed for analysis (Ragin, 2013).

### Case Selection

FsQCA should have a clear hypothetical direction when selecting cases. In this manner, the chosen instances have a degree of similarity. Also, the degree of diversity should be considered in case selection. In the least number of instances, the main criterion should reach the highest degree of “heterogeneity” (Ragin, 2013). Case selection should not be mechanical random sampling in small and medium sample studies (Rihoux and Ragin, 2012). The total homogeneity of the case population is combined with the greatest heterogeneity of the case population (Benford and Ragin, 1996). Therefore, all selected rumor cases have been refuted in the official media to ensure the accuracy of case selection. First, select brand rumors that spread widely, including positive rumors and negative rumors. Second, these rumors have been refuted in mainstream media, including China Internet Joint Rumor Refutation Platform^1^ and BBC News.^2^ Based on the credibility, novelty, and valence of rumors, five experts analyzed and graded the rumors. Credibility refers to the rumors capacity for consumers’ belief. Novelty emphasizes a new or unusual degree of rumors. Valence is the degree to which rumors are positive or negative. Therefore, selecting 2^3^ types of rumors in the last 20 years, covering credibility, novelty, and valence, respectively (Appendix A), has enriched research into the spread of rumors; this has ensured that the investigation of rumors is not limited to the spread of a certain form of rumors.

### Data Collection and Measure

An online survey questionnaire designed by www.wjx.cn was collected from September to December 2020. Respondents filled out the questionnaire anonymously and were allowed to exit the questionnaire at any time as they wished. Of the 547 survey invitations sent, 446 valid survey questionnaires were returned, with an 81% recovery rate. The respondents were mainly from mainland China. Table 1 outlines other demographic features of the study.

**TABLE 1:** Demographic characteristics of the sample.

Negative rumors	Number	Percentage	Positive rumors	Number	Percentage
Gender	208		Gender	208	
Male	105	50.5%	Male	103	49%
Female	103	49.5%	Female	105	51%
Age			Age		
18∼25	39	18.8%	18∼25	48	22.8%
26∼30	83	39.9%	26∼30	83	40.3%
31∼40	67	32.2%	31∼40	56	27.2%
>40	19	9.1%	>40	21	9.7%
Education			Education		
High school	52	25%	High school	52	24.8%
College/Associate degree	63	30.3%	College/Associate degree	66	31.6%
University/Bachelor degree	93	44.7%	University/Bachelor degree	90	43.6%

Briefly, respondents read a piece of information (rumors) randomly and scored the following items based on their judgment on the content of the information. All variables were measured using a 7-point Likert scale: 1 denotes completely disagree, while 7 denotes absolutely agree. The details are as follows.

#### Outcome Factor

##### Information Sharing Intention

The outcome factor is the information sharing intention, “I intend to share the ‘message’ with others frequently.”

#### Cause Conditions of Passively Sharing Rumors Motivation

##### Anxiety

The anxiety management motivation is centered on the fact that people share rumors to alleviate anxiety. There are four items to measure anxiety (Sudhir and Unnithan, 2014).

##### Hope

Hope emotion was measured by four items (Krafft et al., 2019). For example, “I will share this message because it is hopeful for me.”

##### Herd Mentality

Herd mentality is the tendency of individuals to think and conduct in ways that correspond to the group’s norms rather than acting independently, having four items (Song et al., 2019).

##### Trust

Trust is the proclivity of consumers to believe rumors and would like to share them with others. However, distrust rumors are also promote people to share due to alleviate the uncertainty associated with rumors. This was measured by four items (Gefen, 2000; Podsakoff et al., 2003). For instance, “I trust the information (rumors) that I shared to be true.”

#### Cause Conditions of Actively Sharing Rumors Motivation

##### Relationship Management

The focus for relationship management is on the effect of individuals who regard rumors as valuable information for improving relationships. Based on the research of Sudhir and Unnithan (2014), we measure it by four items.

##### Self-Improvement

This is when people share rumors with others or group members to boost their esteem (Bordia and Di Fonzo, 2017). This too can be measured using four items (Sudhir and Unnithan, 2014).

##### Altruism

Altruism refers to a type of motivation that seeks to benefit others rather than one’s interests (Batson, 2010). Apuke and Omar (2021) outlined for different items for measuring altruism.

### Dataset and Calibration

Data calibration, which allocates all variables a value between 0 and 1, is the most critical phase in the fsQCA research method (Ragin, 2013). The fsQCA technology requires that researchers not only make conscious choices but also explain them (Ragin, 2013). The basic criteria for designing fuzzy-sets entails calibrating membership ranking, thus calibration should not be mechanical (Ragin, 2013). Notably, researchers choose how many fuzzy sets to use, depending on study requirements (Ragin, 2013). Given that types of rumors and the corresponding causes are complex, this paper adopts the direct method, with percentile-based qualitative breakpoints, to guarantee the rationality of variable assignment. The percentile is used to complete data calibration, because the data can be biased. Particularly, 90% represents the full in-set membership, whereas 10 and 50% denote full out-set and intermediate set membership, respectively. The value of each variable is calibrated according to the logic function, which is suitable for the above three anchor points integrated into the fsQCA software. A summary of breakpoints is outlined in Table 2. The proportional reduction in inconsistency (PRI) should be higher than 0.70, while most coverage values range from 0.25 to 0.65 (Woodside, 2013; Kraus et al., 2018). Finally, the fsQCA method proposes that the researcher interprets three sets of solutions, namely the complex, parsimonious, and intermediate results. A summary of the variables, calibrations and descriptive statistics is presented in Table 2.

**TABLE 2:** Sets, calibrations and descriptive statistics after calibrations regarding positive and negative rumours.

	Fuzzy-set calibrations			Descriptive statistics					
	Full out	Crossover	Full in	Mean	SD	Min	Max	*N* Cases	Missing
**Positive rumors sets**									
SR	2	4	6	0.588	0.320	0.01	0.99	208	0
Hope	2.667	4.667	6	0.498	0.330	0.01	0.99	208	0
HM	3.25	5.25	6.5	0.486	0.319	0.01	0.99	208	0
Trust	2.5	4.5	6.25	0.516	0.324	0.01	0.99	208	0
RM	2.225	4	5.75	0.488	0.336	0.01	0.99	208	0
SI	3.25	4.75	6.25	0.494	0.369	0.01	0.99	208	0
Altruism	3.5	5	6.5	0.491	0.344	0	0.98	208	0
**Negative rumors sets**									
SR	3	5	7	0.518	0.328	0	0.95	208	0
Anxiety	3	4,667	6	0.481	0.329	0	0.99	208	0
HM	3.25	5	6.5	0.522	0.32	0.01	0.97	208	0
Trust	2.725	4.5	6	0.508	0.335	0.01	0.99	208	0
RM	2	3.75	5.25	0.503	0.33	0.01	0.99	208	0
SI	2.725	4	5.5	0.483	0.362	0	1	208	0
Altruism	3.75	5.25	6.5	0.523	0.357	0	0.99	208	0

*SR, Sharing Rumors; HM, Herd Mentality; RM, Relationship Management; SI, Self-improvement.*

## Results

### Necessary Conditions

Necessity conditions for each variable should be verified before building the truth table. Briefly, necessary condition refers to circumstances that must exist to generate a result, but whose presence does not necessarily cause the result to occur. Necessary conditions for sharing and not sharing positive and negative rumors are outlined in Tables 3, 4. However, the agreement value of all conditions is less than 0.9, suggesting that dissemination of positive and negative rumors does not require necessary conditions. This affirms the need to analyze the effect of conditional configuration on positive and negative rumors.

**TABLE 3:** Outline of necessary conditions required for sharing positive and negative rumors.

Outcomes: Sharing positive rumors			Outcomes: Sharing negative rumors		
Rumors sets	Consistency	Coverage	Rumors sets	Consistency	Coverage
Hope	0.691	0.816	Anxiety	0.705	0.758
∼Hope	0.528	0.617	∼ Anxiety	0.541	0.541
HM	0.695	0.841	HM	0.733	0.727
∼HM	0.531	0.607	∼HM	0.511	0.555
Trust	0.638	0.726	Trust	0.651	0.663
∼Trust	0.568	0.691	∼Trust	0.573	0.604
RM	0.653	0.786	RM	0.694	0.714
∼RM	0.550	0.632	∼RM	0.542	0.565
SI	0.656	0.781	SI	0.632	0.677
∼SI	0.511	0.593	∼SI	0.557	0.558
Altruism	0.671	0.804	Altruism	0.690	0.684
∼ Altruism	0.532	0.615	∼ Altruism	0.524	0.569

*HM, Herd Mentality; RM, Relationship Management; SI, Self-improvement. ∼ means the absence of. For example: ∼ Self-improvement = absence of Self-improvement.*

**TABLE 4:** Configurations for sharing and absence sharing positive rumors.

Sharing positive rumors solutions							Absence sharing positive rumors solutions	
	SPR1a	SPR1b	SPR1c	SPR2	SP3Ra	SPR3b	∼SPR1	∼SPR2
Hope	•	•	⚫	⊗			⊗	⊗
HM		•	•	⚫	⚫	⚫	⊗	⊗
Trust			⊗	•		⊗	⊗	
RM	•	•			•	⊗		•
SI		•		⚫	⚫	⚫	⊗	⊗
Altruism	•		⚫	⚫	⚫	⊗	⊗	⊗
Raw coverage	0.388	0.335	0.293	0.192	0.308	0.177	0.397	0.310
Unique coverage	0.065	0.051	0.046	0.005	0.0224	0.028	0.122	0.035
Consistency	0.949	0.970	0.955	0.940	0.975	0.934	0.907	0.931
Solution coverage: 0.598							Solution coverage: 0.432	
Solution consistency: 0.932							Solution consistency: 0.892	

*HM, Herd Mentality; RM, Relationship Management; SI, Self-improvement; SPR, Sharing Positive Rumors; ∼ means the absence of. For example: ∼ SPR = absence of Sharing Positive Rumors. The large symbols* ⚫ *(present) and* ⊗ *(absent) denote core conditions. The small symbols • (present) and* ⊗ *(absent) denote peripheral conditions. Black spaces indicate “don’t care.”*

### Sufficiency Analysis for Sharing Positive and Negative Brand Rumors

FsQCA findings regarding study configuration, solution coverage, and solution consistency are outlined in Tables 4, 5. The large symbols 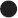 (present) and 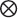 (absent) denote core conditions, whereas small ones 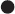 (present) and 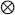 (absent) represent peripheral conditions (Fiss, 2011). Blank spaces indicate Do not care solution. The overall solution coverage shows the degree to which sharing or not sharing positive and negative rumors can be calculated, based on a set of configurations. Overall, the findings show that the total consistency rate that makes people to share or not share positive and negative rumors approaches the agreed threshold of 0.80.

**TABLE 5:** Configurations for sharing and absence sharing negative rumors.

Sharing negative rumors solutions					Absence sharing negative rumors solutions			
	SNR1a	SNR1b	SNR1c	SNR2	∼S NR1	∼S NR2	∼S NR3a	∼S NR3b
Anxiety	⚫	•	•		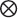	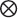	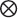	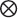
HM		•	⚫	•	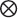		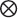	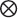
Trust	⚫	•		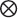	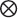	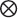	•	•
RM	⚫	•	⚫	•		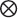	•	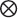
SI	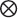		⚫	•	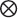	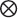		•
Altruism	⚫	•	⚫	•	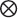	⚫	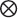	
Raw coverage	0.222	0.304	0.281	0.214	0.304	0.256	0.256	0.252
Unique coverage	0.022	0.001	0.004	0.0248	0.106	0.010	0.0306	0.047
Consistency	0.923	0.909	0.915	0.927	0.927	0.909	0.900	0.900
Solution coverage: 0.396					Solution coverage: 0.551			
Solution consistency: 0.919					Solution consistency: 0.871			

*HM, Herd Mentality; RM, Relationship Management; SI, Self-improvement; SNR, Sharing Negative Rumors; ∼ means the absence of. For example: ∼ SNR = absence of Sharing Negative Rumors. The large symbols* ⚫ *(present) and* 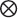 *(absent) denote core conditions. The small symbols* • *(present) and* 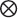 *(absent) denote peripheral conditions. Black spaces indicate “don’t care.”*

### Configurations for Sharing and Absence Sharing Positive Rumors

Three first-level solutions cause people to spread positive rumors, and these have solution coverage and consistency of 0.598 and 0.932, respectively. Firstly, SPR1a, SPR1b, and SPR1c research findings indicate that emotional stimulation of hope plays an important role in spread of positive rumors. However, the likelihood of consumers spreading rumors solely based on hope is low, and requires an interaction with other conditions. For instance, SPR1b results demonstrate that individuals employ relationship management and self-improvement motivations to actively share positive brand rumors. In fact, the likelihood of them sharing these rumors is high, as evidenced by a Raw coverage of 0.335 and a Consistency of 0.970.

Secondly, altruism and the relationship management motivations of egoism do not conflict in the behavior of consumers who spread positive brand rumors (SPR1a and SPR3a), implying that both altruistic and reciprocal behaviors can exist concurrently in personal behavior. This phenomenon, which was confirmed by West et al. (2007), is attributed to the fact that consumers make altruistic judgments for their benefit or act altruistically for mutual benefit in weak altruistic behavior. SPR1a and SPR3b results reflect the altruistic behavior of consumers who spread rumors to improve their chances of developing a long-term relationship. For instance, consumers are likely to share rumors with others when they have hope and are motivated by relationship management and altruism (SPR1a). According to the theory of social contagion, consumers are likely to share positive brand rumors with others (SPR3a) upon influence by herd mentality (core condition), when they encounter relationship management and self-enhancement motivation, or altruism.

Thirdly, consumers are likely to spread positive brand rumors even without stimulation by hope emotions. SPR2 research findings indicate that consumers are likely to spread positive brand rumors under the guise of herd psychology, if they believe them to be true and get a strong motivation to actively spread them (relationship management and self-enhancement motivation).

Fourthly, although trust affects a consumer’s decision to spread positive rumors, we have discovered that they will spread them even if they do not trust them (SPR1c and SPR3b). Specifically, SP1c results indicate that consumers are still willing to share positive rumors with others under the combined influence of altruism and herd psychology to possibly profit the recipients, even if the consumers distrust these rumors when hope stimulates positive emotions in them. However, if the consumer is self-centered and lacks an altruistic spirit, positive brand rumors will continue to spread due to the herd mentality and self-improvement motivation (SPR3b).

There are two configurations for preventing the spread of positive brand rumors, namely Overall coverage = 0.432, and Consistency = 0.892. Study findings indicate that in order to prevent the spread of positive rumors it is critical to dispel consumers’ hope emotions, herd mentality, relationship management motivation, and altruism. Furthermore, ∼SPR1 results indicate that removal of a consumers’ trust in rumors significantly increases the likelihood of spreading positive rumors (Raw coverage = 0.397, Consistency = 0.907). Indeed, relationship management alone cannot subjectively motivate consumers to spread positive brand rumors (∼SPR2).

### Configurations for Sharing and Absence Sharing Negative Rumors

Two first-level solutions, with coverage and consistency values of 0.396 and 0.919, respectively, make individuals to spread negative brand rumors. Firstly, the research findings indicate that emotions also play a significant role in the spread of negative brand rumors (SNR1a, SNR1b, and SNR1c). Notably, negative brand rumors increase consumers’ anxiety, compared to positive ones. However, anxiety-based emotional stimulation alone cannot sufficiently promote the spread of negative brand rumors, but requires a synergistic interaction with other factors. For instance, SNR1b results demonstrate that when negative brand rumors make consumers to experience anxiety emotions and herd mentality, they are likely to believe the rumors, and spread them to others out of altruism (with the aim of preventing harm to the interests of the recipients) and relationship management motivation.

Secondly, coexistence of relationship management motivation and altruism is more pronounced across all paths used to spread negative brand rumors. This means that that negative brand rumors are likely to harm the interests of individuals who buy these brand products. Therefore, consumers may spread negative brand rumors with the aim of not only reducing damage to the interests of other consumers (altruism), but also due to motivation to strengthen their relationship with them. For instance, SNR1a results demonstrate that consumers are likely to promote the spread of negative brand rumors without relying on self-reinforcing motivation when anxiety, relationship management motivation, and altruistic motivation are the primary motivating factors.

Thirdly, in the context of negative brand rumors, this study’s findings also demonstrate that consumers are likely to spread negative brand rumors even if they do not believe in them. Consumers, will spread negative brand rumors (SNR2) due to herd mentality, relationship management, self-enhancement, and altruism. Additionally, negative brand rumors will be spread regardless of whether or not consumers trust the rumor, mainly due to existence of other cause conditions (SNR1c). To prevent the spread of negative brand rumors, three first-level solutions, are suggested. From the study results, it is evident that all configuration paths indicate the importance of reducing anxiety emotion generation. For example, ∼SN1 results demonstrate that, assuming that the motivation for consumer relationship management is irrelevant, limiting the occurrence and effect of other factors can help prevent generation of negative brand rumors. However, this is a difficult prospect in real life. On the other hand, ∼SNR2 results demonstrate that altruistic motivations alone are cannot sufficiently fuel the spread of negative brand rumors. In other words, altruists will not share negative brand rumors if they do not trust them, are not stimulated by anxiety, and lack motivation for relationship management as well as self-improvement. ∼SNR3a and ∼SNR3b results emphasize the importance of anxiety and conformity, even if consumers believe in negative brand rumors. Specifically, ∼SNR3a results demonstrate that reading negative brand rumors do not affect consumers’ anxiety, herd mentality, or altruistic motives, and even if they believe the negative brand rumors and are motivated by relationship management, they may still choose not to spread the rumor. On the other hand, ∼SNR3b results demonstrates that trust and self-improvement motivation alone is insufficient to facilitate the spread of negative brand rumors.

### Robustness Checks

The robustness test is an important part of fsQCA analysis. Specific methods of set and statistical theories are commonly used for robustness testing. Increasing the PRI is a better way to do robustness test. Therefore, we increased the PRI consistency from 0.7 to 0.75, while the configuration of positive and negative rumors sharing remained largely unchanged, respectively, and found that this resulted in a stable outcome. The details in Appendices B,C.

## Discussion, Implications and Conclusion

### Discussion and Conclusion

Numerous studies have solely focused on sources of negative rumors (Buckner, 1965; Fine, 2007; Kapferer, 2013), the conventional rumor transmission pathway before the internet (Caplow, 1947; Buckner, 1965), the integrated rumor pathway model on the internet and how to minimize the negative effect of rumors (Oh et al., 2013; Arif et al., 2016). Although many scholars have studied the spread of rumors and suggested a variety of remedies (Shah and Zaman, 2011; Pan et al., 2018), rumors are still prevalent, a phenomenon that threatens personal, brands and social interests. Collectively, these pieces of evidence indicate that the spread of rumors is a result of a combination of complex factors, and cutting off simply signal transduction may not prevent their spread. Therefore, investigating the spread of rumors, from a multi-dimensional perspective, coupled with the determination of how to prevent their spread, is imperative to effective prevention of the associated negative outcomes.

We investigated psychological motivations that make consumers spread positive and negative brand rumors, then compared similarities and differences between configurations that cause this spreading. Results indicated that emotional stimulation plays a critical role in the spread of both positive and negative rumors. Specifically, positive and negative rumors primarily arouse a consumer’s feelings of hope and anxiety, respectively. In addition, we found that altruism and relationship management motivation (self-interest) can coexist, whether the rumors are positive or negative. This indicates that multiple influencing factors need to be considered when analyzing motivations for the dissemination of brand rumors because the seemingly contradictory antecedents may aid in the spread of brand rumors. Furthermore, our results demonstrated that consumers continue to spread positive and negative brand rumors even though they do not trust them, indicative of the propagation characteristics of uncertain information, according to social contagion theory. Finally, the distinction between paths used to spread positive and negative brand rumors is largely determined by the conditions controlling each configuration (see the “Result” section).

### Theoretical Implication

Firstly, this research discusses the psychological factors that make people to spread brand rumors. These include an individual’s passive and active psychological motivation, personal trust perception, as well as how to prevent the spread of brand rumors using a combination of the above factors. Unlike previously fragmented research, this study delves deeply into the correlation and combination of numerous factors, such as active rumor sharing and passive psychological stimulation, thereby enriching research on rumor spreading. Our findings indicate that numerous factors, and their combinations, contribute to the spread of positive and negative brand rumors. Notably, these causes are complex, and a single one may not effectively contribute to the spread of a crazy rumor.

Secondly, this research contributes to the development of a new theoretical perspective on consumer behavior and the spread of rumors. This study compares the combinations of the main psychological motivations that different rumor properties incite people to spread them, revealing the heterogeneity of the psychological stimuli that confront people with different rumor properties. Moreover, based on social cognition theory, brand rumor sharing is the result of a combination of group and individual psychology, and it is difficult for a single positive or negative motivation to drive people to spread rumors.

Third, with regards to the methodology, previous studies have analyzed pure logical discussion, experiment, structural equation modeling or multiple regression analyses, focusing on one or more dependent variables with a significant influence and net-effect on various main factors. However, these analyses have completely ignored causal linkage and interdependence between variables (Woodside, 2013). In reality, wild rumors are generated by a combination of several factors, and each element has the different effect. The present study is the first report on the use of fsQCA to describe psychological motivation of rumor spreading from a set theory standpoint. Our results not only reveal the combination of factors that affect positive and negative rumor spreading but also demonstrate the disparity between the combinations. These findings are expected to provide valuable solutions to prevent the spread of positive and negative rumors.

### Practical Implications

When faced with rumors against their brands, companies will issue a statement to dispel these rumors. However, brand rumors will continue to spread if the company’s clarification statement is ineffective. Although eliminating uncertainty is one of the most effective ways to combat positive and negative rumors, full eradication of uncertainties in the short term is difficult, necessitating identification of additional methods. Results from the present study indicate that stopping the spread of rumors requires blocking many factors. Consequently, we have suggested different pathways for stopping or slowing down this spread. Notably, positive or negative rumors that can potentially hurt the brands or customers’ long- or short-term interests, should be taken seriously.

Businesses often ignore positive brand rumors, that are beneficial to sale in the short term but could damage the value of the brand in the long term. These should be treated more cautiously. Brands may gain a lot of sales and profits in a short period. However, if the positive rumors are proven to be false, they are likely to cause significant damage to the brand, by causing loss of consumers’ trust and harming brand reputation. When faced with positive rumors, companies should quickly and widely make comprehensive statements to crush the false expectations generated by these rumors, and gain customer trust in the brand as soon as possible. In addition, distributing real information to fight rumors, by taking advantage of the psychology of some customers who share the information with the aim of boosting their impact and close relationships with others, is imperative to limiting damage. Companies need to broadly spread the correct information to prevent rumors from stimulating consumers’ herd conformity.

Negative brand rumors are sometimes detrimental to companies. When faced with interference from negative rumors, brands are advised to react swiftly using a variety of steps to minimize spread and the associated losses. Reducing the anxiety caused by rumors has become a key focus for companies, mainly because negative rumors trigger fear among consumers. Rather than denying that the rumors have nothing to do with the brand, companies need to debunk them by showing that these rumors are false. Notably, several rumors which are not based on fear may still be widely circulated among consumers, mainly because this certain customer enjoy following the public’s choices. For instance, since consumers who have a herd mentality tend to believe in public opinions, encouraging and promoting the right behavior to minimize herding effect represents another way to squash rumors. Besides, sincere statements, as opposed to rigid official ones, can enhance consumers’ trust. This is because they boost a consumers’ willingness to share information, although this time correct information is used to repel rumors.

### Limitations and Future Research

This study had some limitations. Firstly, although we identified factors that promote spread of positive and negative rumors, and the pathways that slow down these pathways, this classification only helps marketers to cope with various types of rumors but does not measure the effect of positive and negative rumors in detail. Further research is needed to determine the effect of spreading positive or negative rumors on a consumer’s purchasing intention or loyalty. Secondly, we did not examine the time dimension, despite the fact that consumers are likely to have altered the path of positive or negative brand rumors over time. Further explorations are needed to ascertain the configuration pathways of positive or negative brand rumors from the rumor’s life cycle standpoint. Future studies are needed to verify numerous brand rumors across different industries, as well as the effect of combining fsQCA with other research methods.

## Data Availability Statement

The raw data supporting the conclusions of this article will be made available by the authors, without undue reservation.

## Ethics Statement

Ethical review and approval was not required for the study on human participants in accordance with the local legislation and institutional requirements. Written informed consent from the patients/participants was not required to participate in this study in accordance with the national legislation and the institutional requirements.

## Author Contributions

XZ and HZ contributed to the conception and design of the study. CX organized the database. YH performed the statistical analysis. XZ wrote the first draft of the manuscript. All authors contributed to manuscript revision, read, and approved the submitted version.

## Conflict of Interest

The authors declare that the research was conducted in the absence of any commercial or financial relationships that could be construed as a potential conflict of interest.

## Publisher’s Note

All claims expressed in this article are solely those of the authors and do not necessarily represent those of their affiliated organizations, or those of the publisher, the editors and the reviewers. Any product that may be evaluated in this article, or claim that may be made by its manufacturer, is not guaranteed or endorsed by the publisher.

## Footnotes

1
http://www.piyao.org.cn/

2
https://www.bbc.com/

## Appendix

**Appendix A:** Rumor messages used in this study (Brands have been masked).

No.	Message	Credibility	Novelty	Valence	Product
1.	While many people are unfamiliar with camel milk, it has long been regarded as an irreplaceable nutritional commodity in a number of countries. Recently, a Kenyan camel milk company teamed up with the Institute of Medicine to conduct research on the role of camel milk in the prevention and treatment of diabetes and coronary artery disease. Camel milk is very similar to human breast milk, and milk contains several immunologically active protein factors. Camel milk contains three times the vitamin D found in milk. Camel milk contains more than three thousand times the amount of insulin found in milk. Then, an analysis of the components of camel milk reveals that it contains a number of hypoglycaemic factors that can help regulate diabetes-related blood sugar levels.	High	High	Positive	Milk
2.	Breakfast is the most vital meal of the day. Oatmeal is a popular breakfast choice for many people. They believe they are nourishing and safe. However, there have been reports recently that the glyphosate content of 26 international oat brands’ products exceeds the standard and may cause cancer if consumed in large amounts for an extended period of time.	High	High	Negative	Oat
3.	After washing rice, many individuals pour out the rice-washing water, but in fact, rice-washing water contains many nutrients and is a natural and safe detergent! Rice-washing water can enable the skin to cleanse and eliminate oil and whitening effects. In rice water, rice bran oil is rich in vitamins B and E, which are capable of protecting the skin against ultraviolet radiation and preventing the development of melanin to whiten the skin.	Low	Low	Positive	Rice Water Cosmetic
4.	Not only do some middle-aged and elderly people have gout today, but many young people have gout sickness. Gout is painful. Gout originates in the human body from the accumulation of uric acid, and uric acid is a purine metabolite. High-protein diets have higher levels of purine and are not ideal for patients with gout. Patients with gout need to be very careful about their diet. Many people claim that soy products are very high in nutritional value, but it contains a lot of calcium. Gout patients should not eat more because tofu contains a certain amount of purine, after eating, gout patients may be more harmful to the body.	Low	Low	Negative	Beans
5.	Recently, an article reported that a 14-year-old girl frequently ate breakfast bread from Brand A and had bowel cancer. Children often eat breakfast bread, according to physicians, excessive consumption of fine grains can affect nutrition and cause cancer. The little bread is in truth, full of poison! First, a lot of “carcinogenic” saccharin is contained in sweet noodles. Some studies suggest that excessive saccharin can cause cancer, and it is not good for human health to consume significant quantities of this synthetic chemical in the long term. Second, the risk of cardiovascular disease increases with emulsifiers.	Low	High	Negative	Food Additives
6.	People spend more time looking at the cell phone screen in the age of smartphones, but mobile phone displays, LEDs, and computer screen lights all create a lot of blue light that will damage the eyes and hands of people, trigger brown pigments, and make skin Macular spots and freckles will deepen the degree of myopia and cause visual fatigue. It is not conducive to normal sleep either. Scientific research has shown that the blue light emitted by electronic product screens can influence users’ eye health, especially causing childhood and adolescent myopia. To achieve the successful blocking of blue light, the blue light cell phone film adopts blue light blocking technology to absorb and transform blue light. It can effectively block ultraviolet rays, short-wave blue rays, soft and dazzling stimulation of the light source.	Low	High	Positive	Anti-blue Glasses
7.	The grape seed extract is a nutritious food that is processed from effective active nutrients derived from vitamin E and other key raw materials from natural grape seeds. The extract of grape seed is a pure natural product and one of the most effective antioxidants found so far from plant sources. Tests have shown that it has 30-50 times the antioxidant effect of vitamin C and vitamin E. There are anti-aging properties of grape seed extract.	High	Low	Positive	Anti-aging drugs
8.	Formaldehyde, pungent odor, colorless gas, scratching human eyes and nose. Its irritation of the skin and mucous membranes is the main risk of formaldehyde. Formaldehyde is a toxic virgin substance that when inhaled in high concentrations, can bind to proteins, and cause severe respiratory tract irritation, oedema, eye irritation, and headache. Allergic dermatitis, stains, and necrosis may be caused by direct skin contact with formaldehyde.	High	Low	Negative	For maldehyde

**Appendix B:** Robustness checks of sharing and absence sharing positive rumors.

Sharing positive rumors solutions							Absence sharing positive rumors solutions

	**SPR1a**	**SPR1b**	**SPR1c**	**SPR2**	**SPR3a**	**SPR3b**	**∼SPR1**
Hope							
HM							
Trust							
RM							
SI							
Altruism							
Raw coverage	0.388	0.335	0.293	0.260	0.192	0.308	0.397
Unique coverage	0.065	0.037	0.020	0.013	0.013	0.022	0.397
Consistency	0.949	0.970	0.955	0.970	0.940	0.975	0.907

Solution coverage:0.583							Solution coverage:0.397
Solution consistency:0.936							Solution consistency:0.907

*HM, Herd Mentality; RM, Relationship Management; SI, Self-improvement; SPR, Sharing Positive Rumors; ∼ means the absence of. For example: ∼ SPR, absence of Sharing Positive Rumors. The large symbols*  *present) and*  *(absent) denote core conditions. The small symbols*  *(present) and*  *(absent) denote peripheral conditions. Black spaces indicate “don’t care.”*

**Appendix C:** Configurations for sharing and absence sharing negative rumors.

Sharing negative rumors solutions			Absence sharing negative rumors solutions		

	**SNR1a**	**SNR1b**	**∼SNR1**	**∼SNR2**	**∼SNR3**
Anxiety					
HM					
Trust					
RM					
SI					
Altruism					
Raw coverage	0.222	0.281	0.306	0.255	0.210
Unique coverage	0.089	0.147	0.118	0.062	0.0536
Consistency	0.923	0.915	0.926	0.924	0.932

Solution coverage:0.396			Solution coverage:0.442		
Solution consistency:0.919			Solution consistency:0.915		

*HM, Herd Mentality; RM, Relationship Management; SI, Self-improvement; SNR, Sharing Negative Rumors;* ∼ *means the absence of. For example:* ∼ *SNR = absence of Sharing Negative Rumors. The large symbols*  *(present) and*  *(absent) denote core conditions. The small symbols*  *(present) and*  *(absent) denote peripheral conditions. Black spaces indicate “don’t care.”*
