# Corrigendum: Sharing or Not: Psychological Motivations of Brand Rumors Spread and the Stop Solutions

**DOI:** 10.3389/fpsyg.2022.935802

**Published:** 2022-07-01

**Authors:** Xu Zhang, Hong Zhu, Yu Huang, Chunqu Xiao

**Affiliations:** ^1^Business School, Nanjing University, Nanjing, China; ^2^Business Administration School, Shanxi University of Finance and Economics, Taiyuan, China

**Keywords:** brand rumors, Consumer Cognition, sharing rumors motivation, Social contagion theory, fsQCA

In the original article, there was a mistake in two of the author's names. Two author names were incorrectly spelled as “Yu Hwang” and “Chuqu Xiao.” The correct spelling is “Yu Huang” and “Chunqu Xiao.” The original article has been updated.

In the original article, there was a mistake in the title of “Table 2.” The title was originally, “**Table 2**. Sets, calibrations and descriptive statistics regarding positive and negative rumors.” But should be “**Table 2**. Sets, calibrations and descriptive statistics after calibrations regarding positive and negative rumours.” The corrected Table 2 appears below.

**Table 2:** Sets, calibrations and descriptive statistics after calibrations regarding positive and negative rumours.

	**Fuzzy-set calibrations**			**Descriptive statistics**					
	**Full out**	**Crossover**	**Full in**	**Mean**	**SD**	**Min**	**Max**	***N* cases**	**Missing**
**Positive rumors sets**									
SR	2	4	6	0.588	0.320	0.01	0.99	208	0
Hope	2.667	4.667	6	0.498	0.330	0.01	0.99	208	0
HM	3.25	5.25	6.5	0.486	0.319	0.01	0.99	208	0
Trust	2.5	4.5	6.25	0.516	0.324	0.01	0.99	208	0
RM	2.225	4	5.75	0.488	0.336	0.01	0.99	208	0
SI	3.25	4.75	6.25	0.494	0.369	0.01	0.99	208	0
Altruism	3.5	5	6.5	0.491	0.344	0	0.98	208	0
**Negative rumors sets**									
SR	3	5	7	0.518	0.328	0	0.95	208	0
Anxiety	3	4.667	6	0.481	0.329	0	0.99	208	0
HM	3.25	5	6.5	0.522	0.32	0.01	0.97	208	0
Trust	2.725	4.5	6	0.508	0.335	0.01	0.99	208	0
RM	2	3.75	5.25	0.503	0.33	0.01	0.99	208	0
SI	2.725	4	5.5	0.483	0.362	0	1	208	0
Altruism	3.75	5.25	6.5	0.523	0.357	0	0.99	208	0

*SR, Sharing Rumors; HM, Herd Mentality; PM, Relationship Management; SI, Self-improvement*.

The authors apologize for this error and state that this does not change the scientific conclusions of the article in any way. The original article has been updated.

## Publisher's Note

All claims expressed in this article are solely those of the authors and do not necessarily represent those of their affiliated organizations, or those of the publisher, the editors and the reviewers. Any product that may be evaluated in this article, or claim that may be made by its manufacturer, is not guaranteed or endorsed by the publisher.

